# Supplementary material for: An IgG‐based bispecific antibody for improved dual targeting in PSMA‐positive cancer
Source: EMBO Mol Med. 2020 Dec 29;13(2):e11902. doi: 10.15252/emmm.201911902 (PMC7863392; doi:10.15252/emmm.201911902)
Supplement: Supplementary file 1 — Expanded View Figures PDF [file EMMM-13-e11902-s001.pdf]

Expanded View Figures

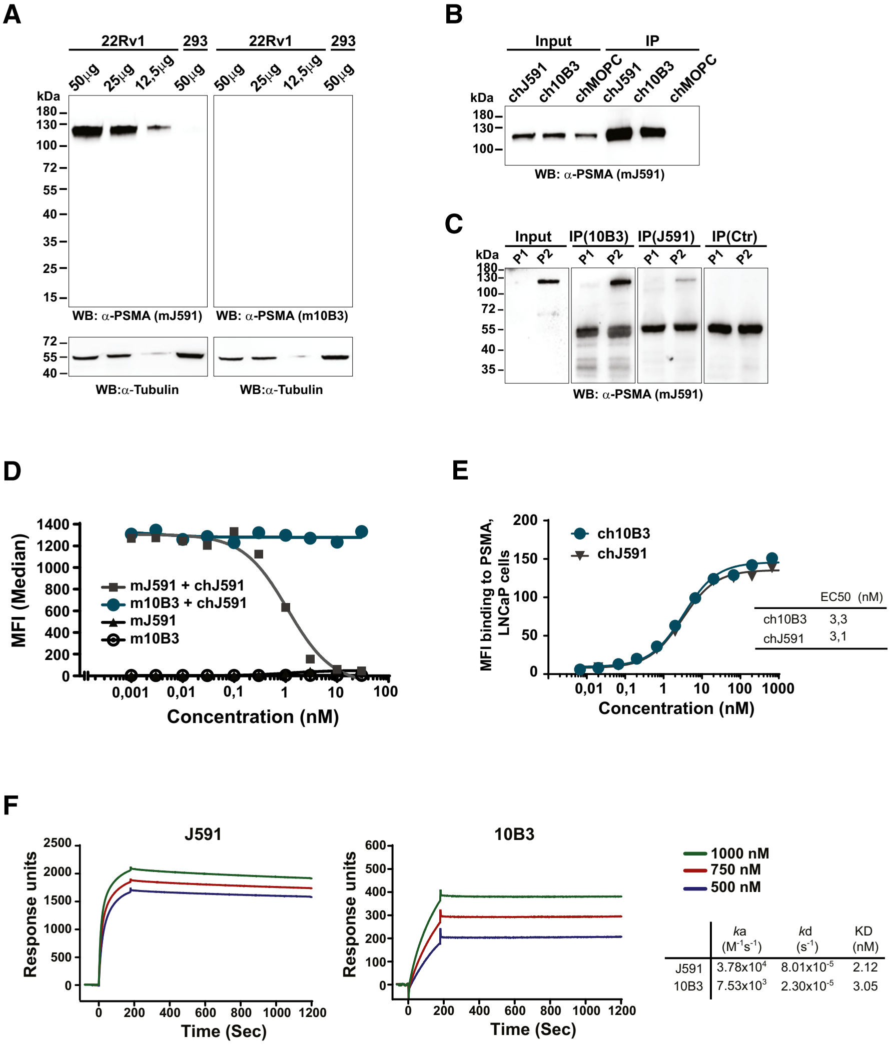

Figure EV1. ***In vitro* characterization of the PSMA antibodies 10B3 vs J591.**

A Western blot analysis was performed using the indicated amounts of protein extracts prepared from 22Rv1^{high} cells or from PSMA-negative HEK-293 cells. Blots were analyzed using anti-PSMA (mJ591 or m10B3) and anti-tubulin as a loading control.

B, C PSMA protein was immunoprecipitated from 22Rv1^{high} cells (B) and lung SCC samples (C) using chimeric J591, 10B3, and MOPC-21 (control) antibodies. The inputs (5%) and immunoprecipitates (30%) were analyzed by Western blot using murine J591 antibody.

D Mouse Sp2/0 cells transfected with human PSMA were incubated with the indicated concentrations of primary murine mAbs (mJ591, m10B3), followed by a chimeric J591 antibody (chJ591, 10μg/ml) and finally with fluorescence labeled mouse anti-human antibody for specific detection of chJ591 as indicated.

E, F Chimeric versions of both mAbs were bound to LNCaP cells (E) and to a protein A coated sensor chip (F) and analyzed by flow cytometry and SPR, respectively, as described in the Materials and Methods section.

Source data are available online for this figure.

© 2021 The Authors

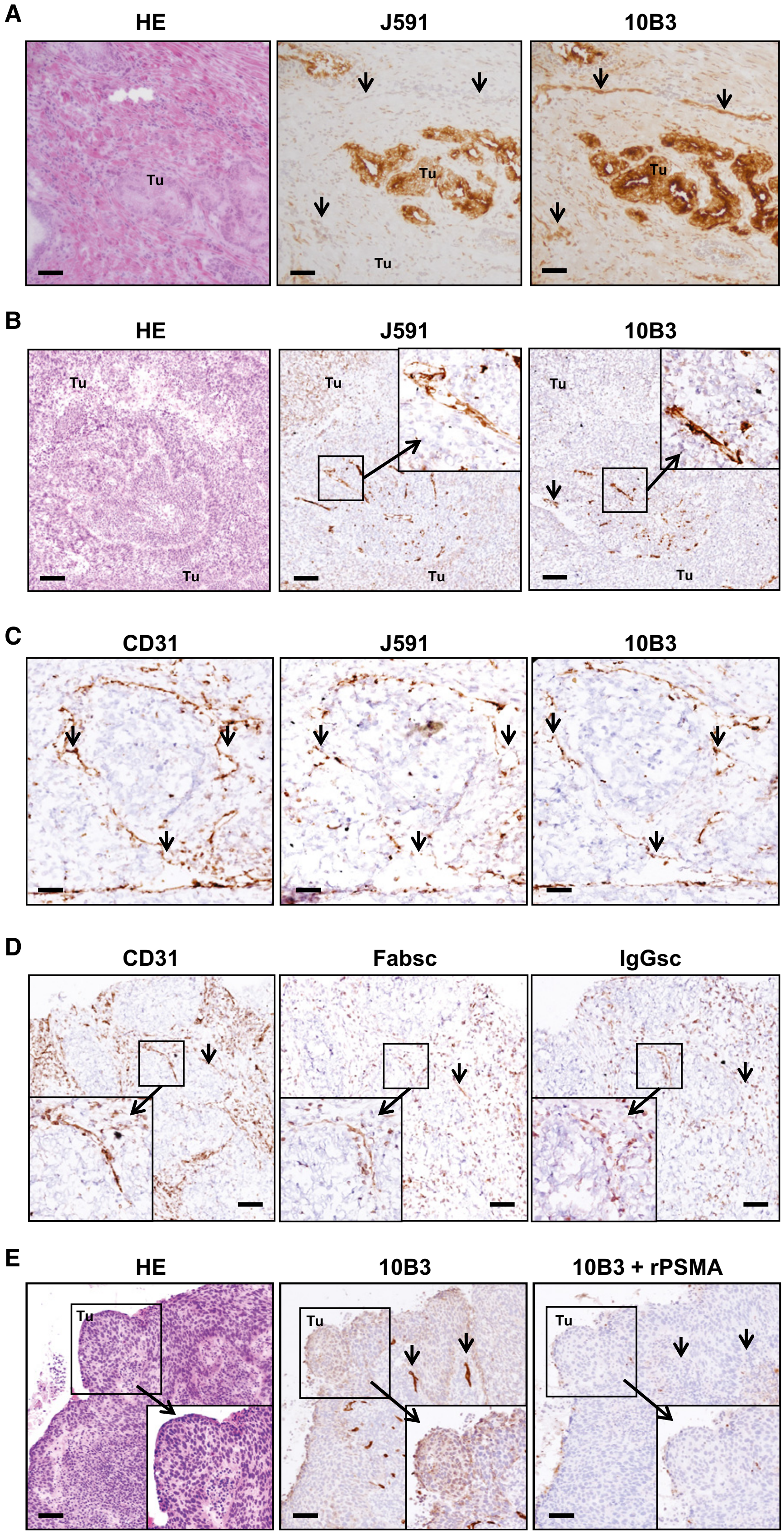

Figure EV2. Staining of exemplary prostate carcinoma and lung SCC samples.

A Directly consecutive 3-µm sections obtained from prostate carcinoma samples were stained with 10B3 and J591 mAbs. Arrows point to vessels. Tu: tumor. HE: Hematoxylin/Eosin staining. Scale 30 µm.

B–E Directly consecutive 3-µm sections (obtained from the same lung SCC sample for each antibody panel) were analyzed by immunohistochemistry as described in the Materials and Methods section. Note that in (C, D) a tumor sample with predominant vascular expression of PSMA (cancer cells PSMA-very low intensity) was chosen to facilitate assessment of vascular staining. (B) Binding of murine 10B3 or J591 mAbs. Arrows point to vessels. Tu: tumor. HE: Hematoxylin/Eosin staining. Scale 30 µm. (C) Staining with 10B3, J591, or anti-CD31 as marker for vessels. Arrows point to vessels. Scale 10 µm. (D) Binding of anti-CD31 as well as biotinylated IgGsc and Fabsc molecules. Note that staining intensity with bsAbs is lower compared to CD31 due to the necessity to utilize a different detection protocol, and bsAb may additionally bind to tumor-infiltrating T cells, resulting in differential staining patterns. Arrows point to vessels. Scale 20 µm. (E) Staining with 10B3 in the absence or presence of 50 µg recombinant PSMA protein. HE: Hematoxylin/Eosin staining. Scale 40 µm.

Source data are available online for this figure.

© 2021 The Authors

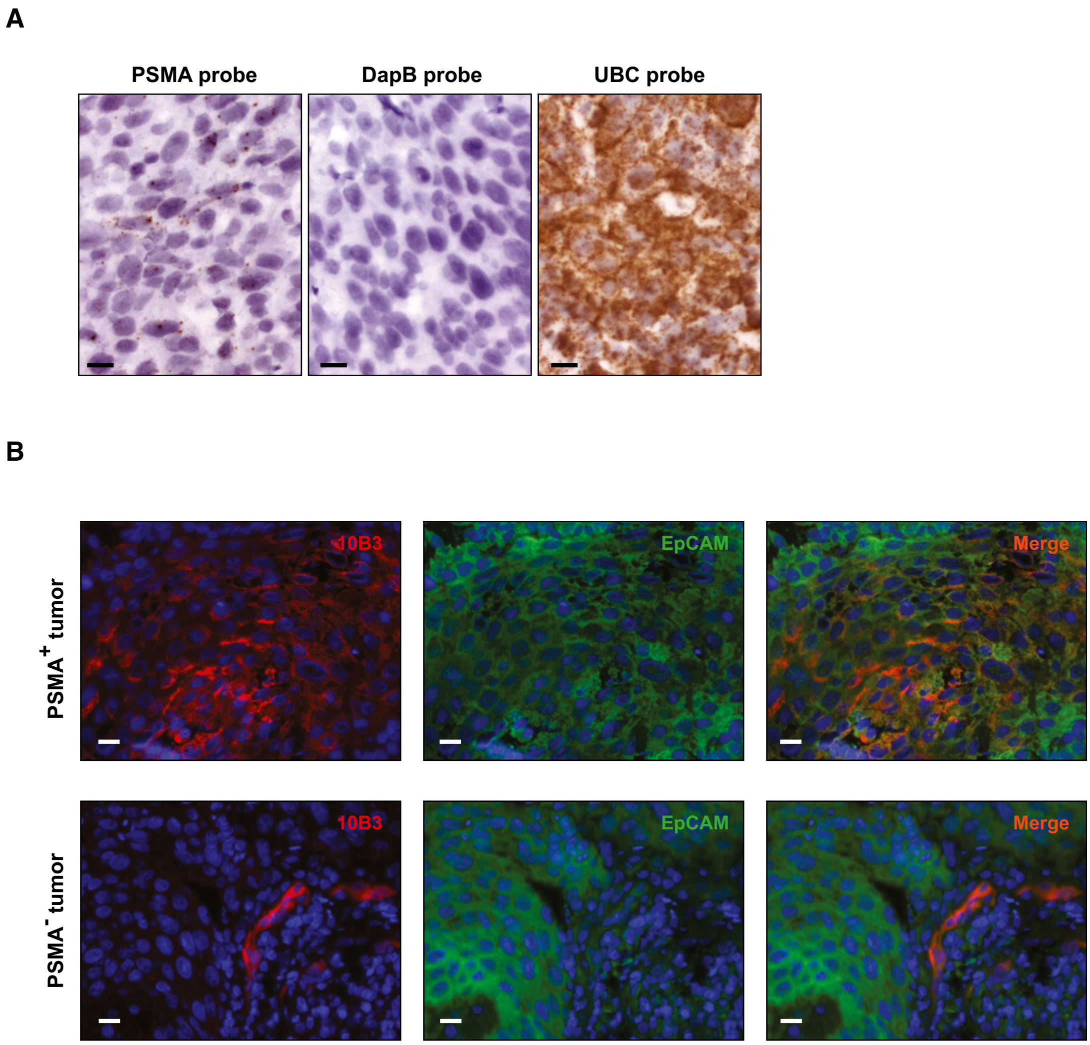

Figure EV3. PSMA RNA expression and membrane staining on lung SCC cryosections.

A RNA *in situ* hybridization was performed using the RNAscope® kit and probes against PSMA, DapB (negative control), and UBC (positive control) as described in the Materials and Methods section. Scale 10 μm.

B Lung SSC tumor sections, (upper panel with PSMA-positive tumor lower panel with PSMA-negative tumor), were stained with 10B3 and a Cy3-labeled secondary antibody followed by a FITC-labeled antibody to the human EpCAM molecule. Scale 10 μm.

© 2021 The Authors

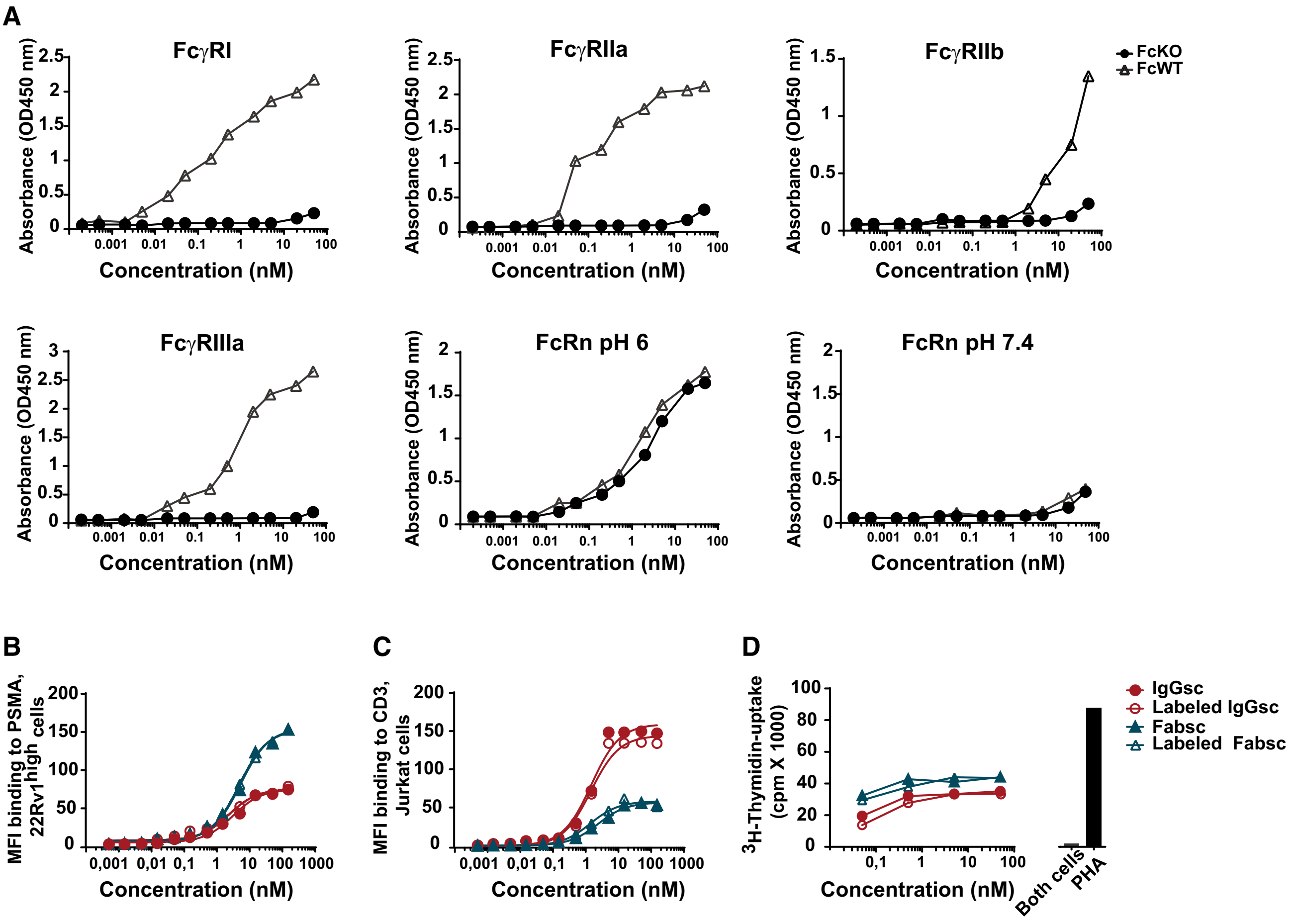

Figure EV4. Consequences of Fc silencing and chelation of CC-1.

A Binding of CC-1 (FcKO) and a variant containing a wild type Fc part (FcWT) to the indicated his-tagged FcR was determined by ELISA. All experiments were performed at a neutral pH except for FcRn, where binding was additionally evaluated at a pH of 6. Means of duplicate measurements are shown.

B, C BsAbs in the Fabsc and IgGsc format chelated or not to p-NCS-Bn-NODAGA were incubated with (B) 22Rv1^{high} (PSMA^{+}) cells or (C) Jurkat (CD3^{+}) cells followed by binding analysis by flow cytometry.

D LNCaP cells were incubated with PBMC and the indicated concentrations of chelated or native bsAbs, and T-cell activation was assessed using a ^{3}H thymidine uptake assay.

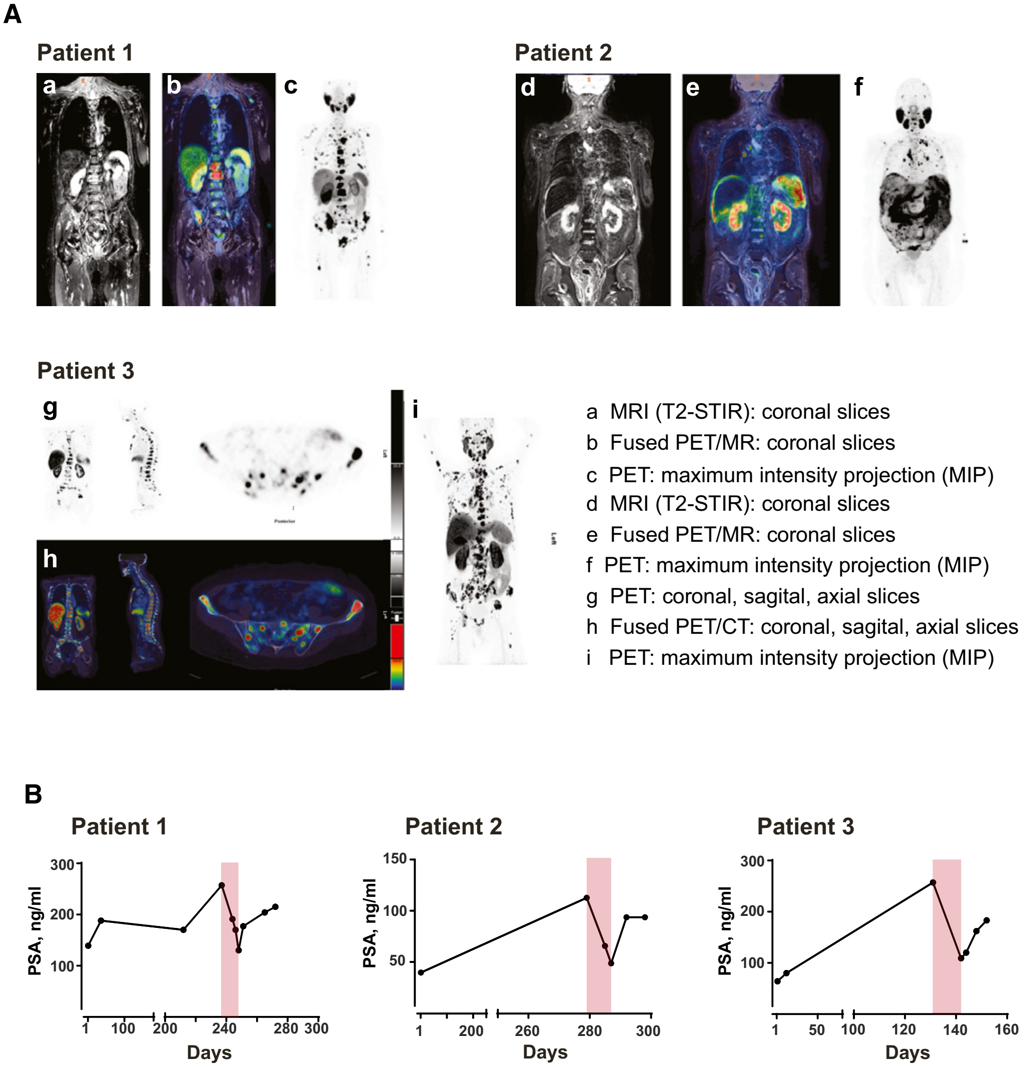

Figure EV5. PET scans and long-term PSA levels of the three treated patients with castrate resistant, metastasized prostate carcinoma.

A Pre-therapeutic PSMA expression of metastatic prostate cancer lesions was visualized by means of contrast enhanced whole body multiparametric PET/MRI (patient 1 and 2) or PET/CT (patient 3). Image data were acquired 60 min after i.v. injection of [^{18}F]-PSMA-1007 (250–325 MBq), a labeled peptide tracer for PET imaging that specifically binds to PSMA. It is used according to §13.2B AMG (German drug law) for PSMA-PET which has become standard clinical care in Germany. Representative images of the patients are shown: patient 1 presented with osseous and lymphonodal metastases with intense PSMA expression (a–c); patient 2 suffered from PSMA-expressing peritoneal carcinomatosis, multiple PSMA-expressing bone and lymph node metastases (d–f); in patient 3, osseous and lymphonodal metastases as well as local recurrence with intense PSMA expression are detected (g–i).

B Long-term PSA values monitored prior, during (highlighted in light red), and after CC-1 therapy. After documented failure of established treatment, patients were free of disease-specific therapy for at least 4 weeks prior to application of CC-1.

© 2021 The Authors
