# Supplementary figures and images for: An IgG‐based bispecific antibody for improved dual targeting in PSMA‐positive cancer
Source: EMBO Mol Med. 2020 Dec 29;13(2):e11902. doi: 10.15252/emmm.201911902 (PMC7863392; doi:10.15252/emmm.201911902)

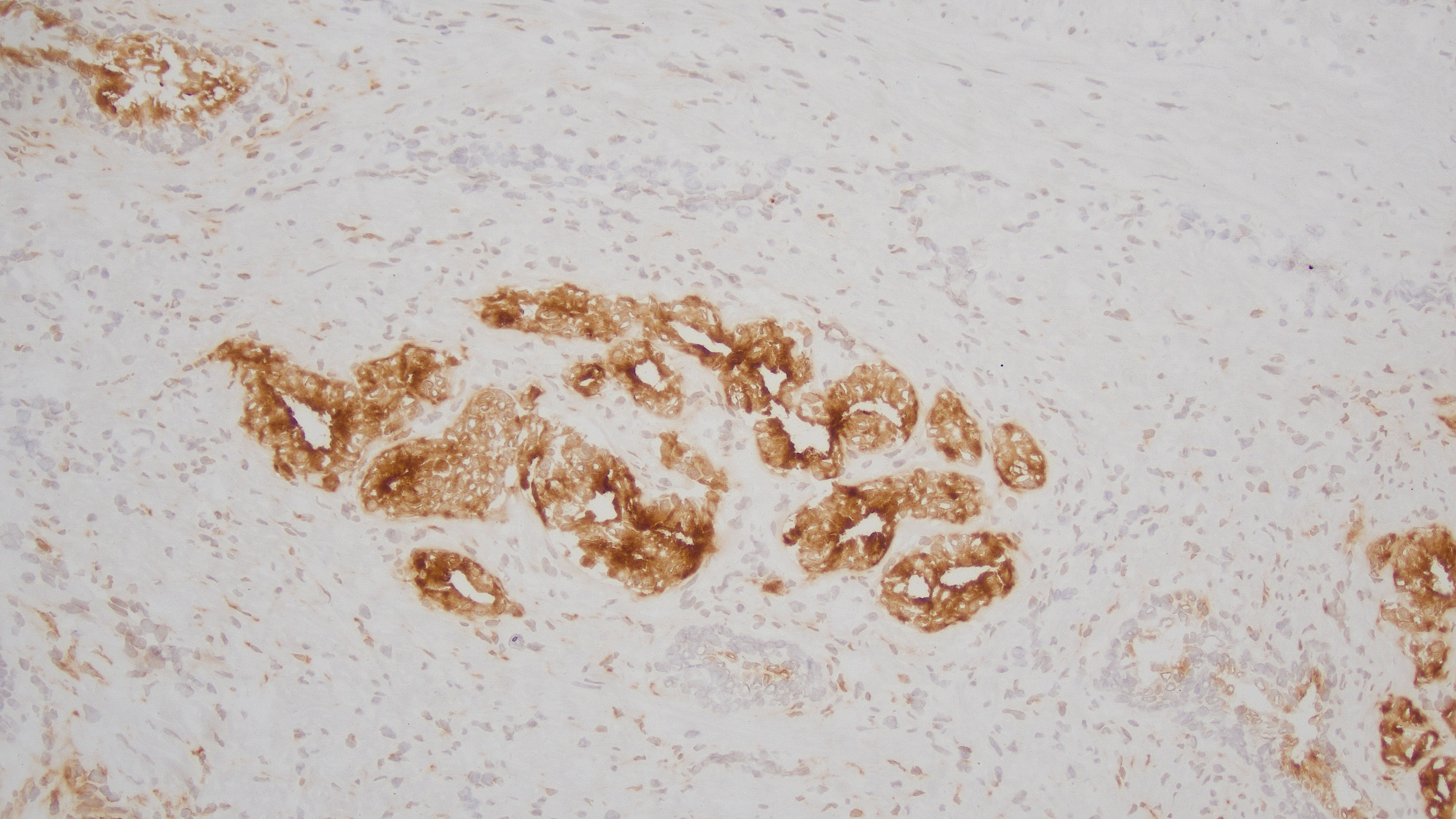

Supplement: Supplementary file 2 — Source Data for Expanded View [file EMMM-13-e11902-s002.zip › EMM-2019-11902_V3_SourceDataForEV2A-E/FigEV2A_J591.jpg]

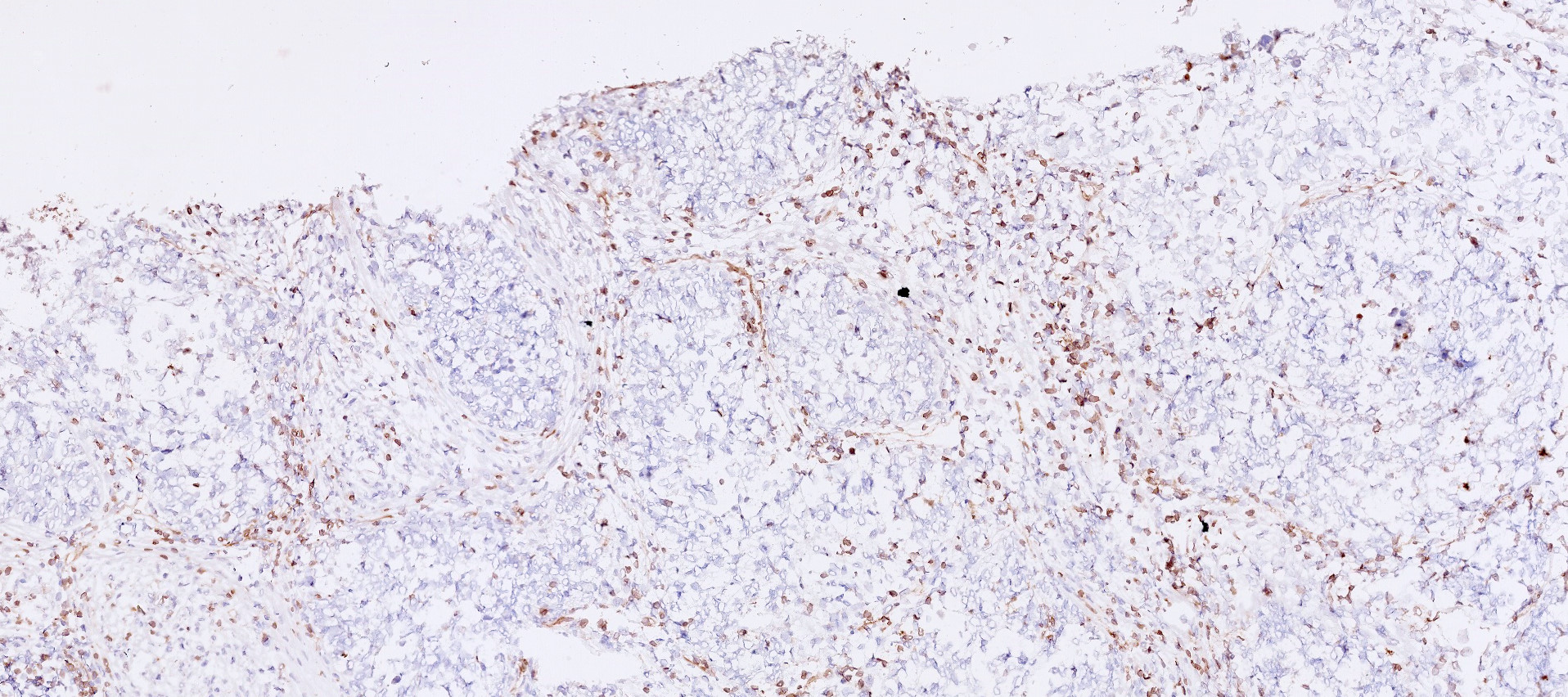

Supplement: Supplementary file 2 — Source Data for Expanded View [file EMMM-13-e11902-s002.zip › EMM-2019-11902_V3_SourceDataForEV2A-E/FigEV2D_Fabsc.bmp]

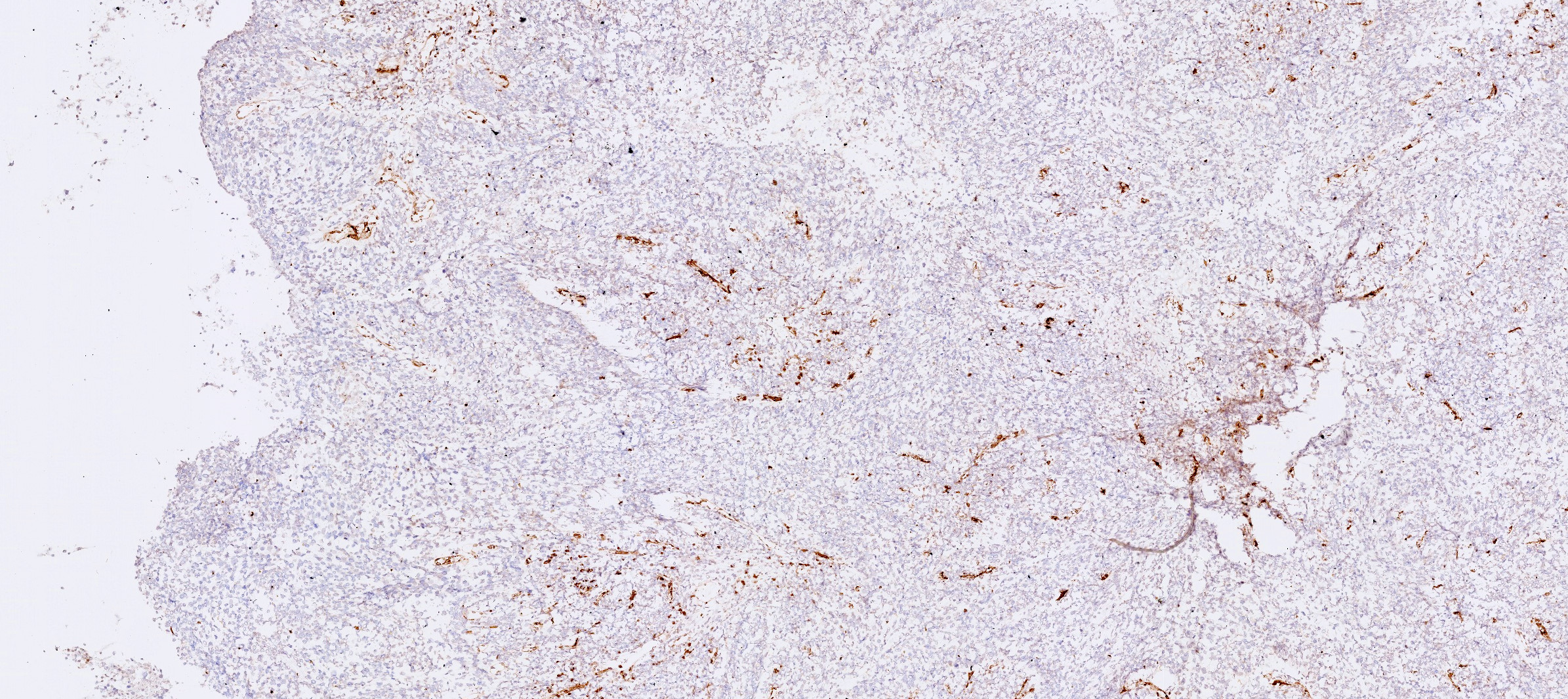

Supplement: Supplementary file 2 — Source Data for Expanded View [file EMMM-13-e11902-s002.zip › EMM-2019-11902_V3_SourceDataForEV2A-E/FigEV2B_J591.bmp]

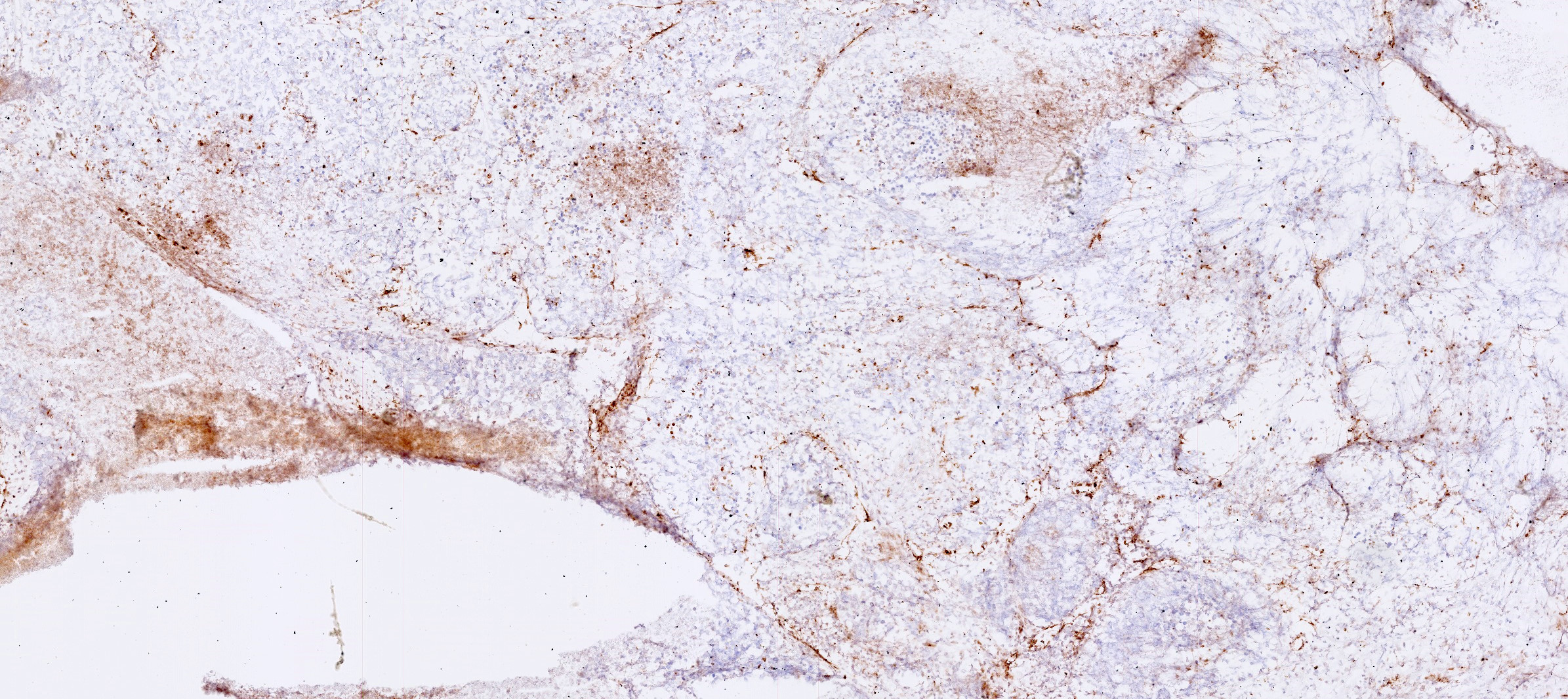

Supplement: Supplementary file 2 — Source Data for Expanded View [file EMMM-13-e11902-s002.zip › EMM-2019-11902_V3_SourceDataForEV2A-E/FigEV2C_J591.jpeg]

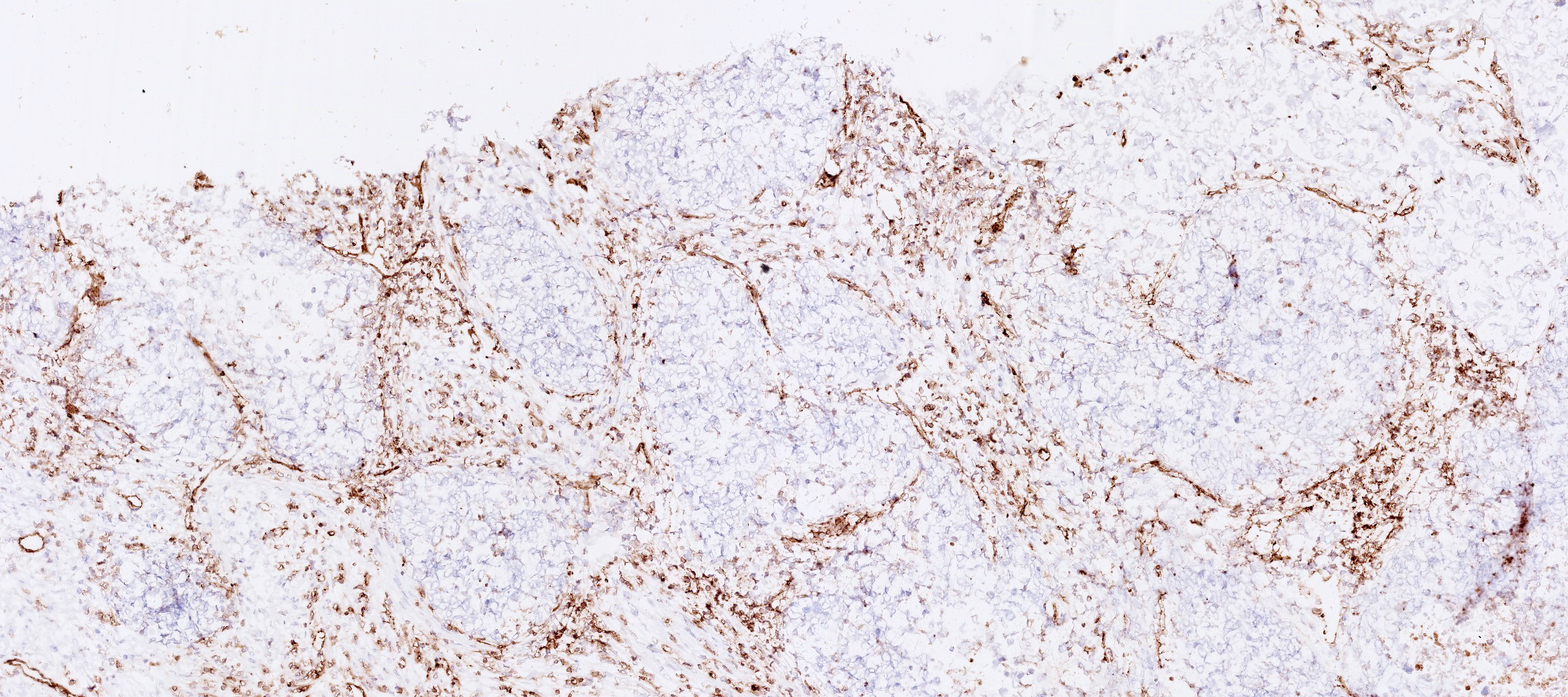

Supplement: Supplementary file 2 — Source Data for Expanded View [file EMMM-13-e11902-s002.zip › EMM-2019-11902_V3_SourceDataForEV2A-E/FigEV2D_CD31.bmp]

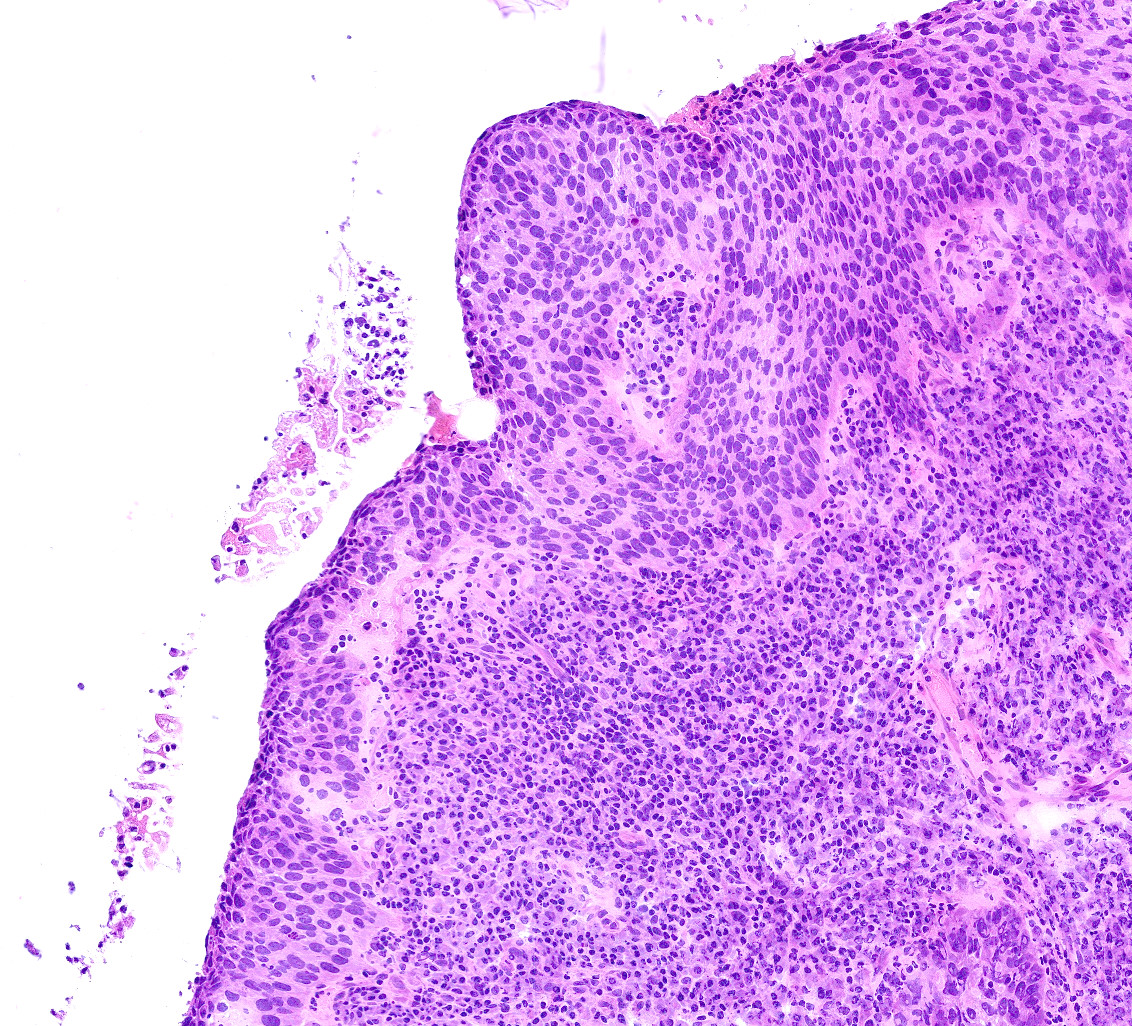

Supplement: Supplementary file 2 — Source Data for Expanded View [file EMMM-13-e11902-s002.zip › EMM-2019-11902_V3_SourceDataForEV2A-E/FigEV2E_HE.bmp]

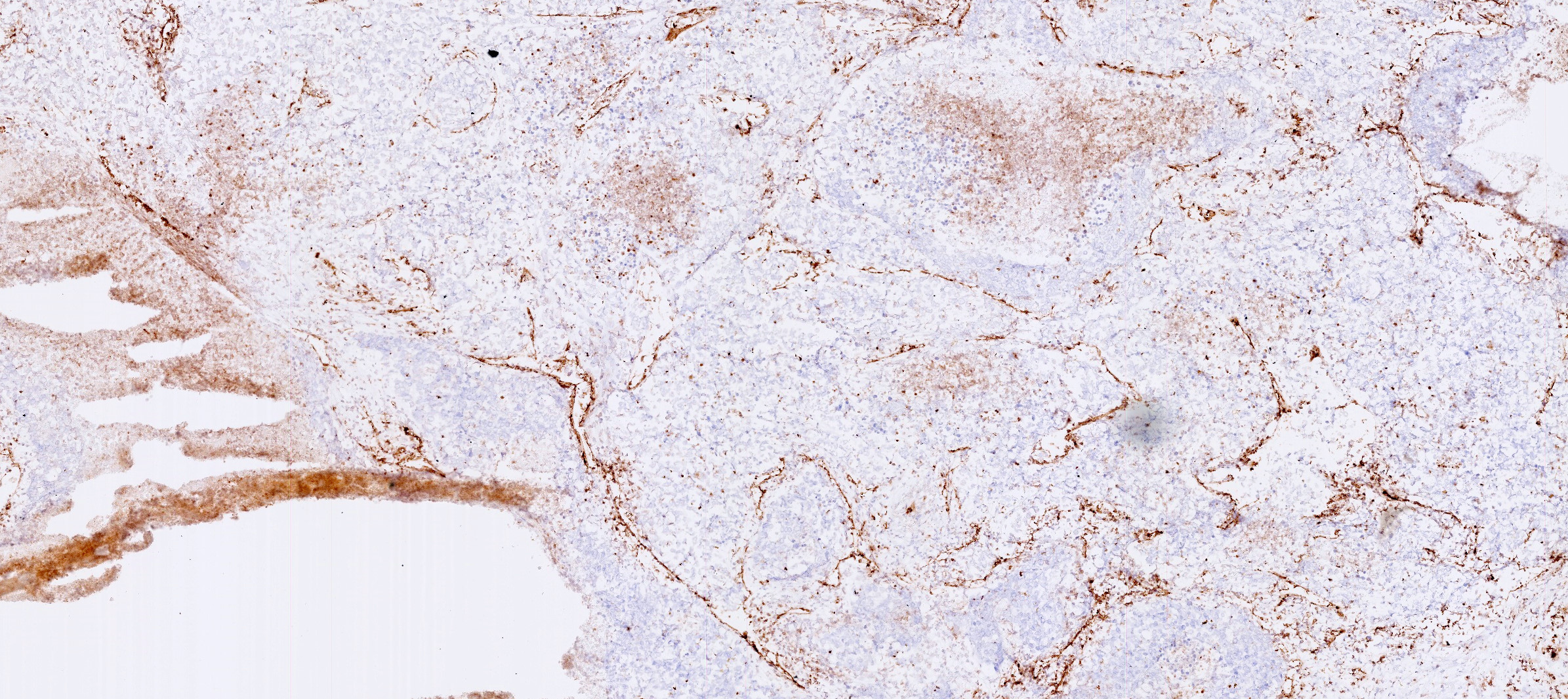

Supplement: Supplementary file 2 — Source Data for Expanded View [file EMMM-13-e11902-s002.zip › EMM-2019-11902_V3_SourceDataForEV2A-E/FigEV2C_CD31.jpeg]

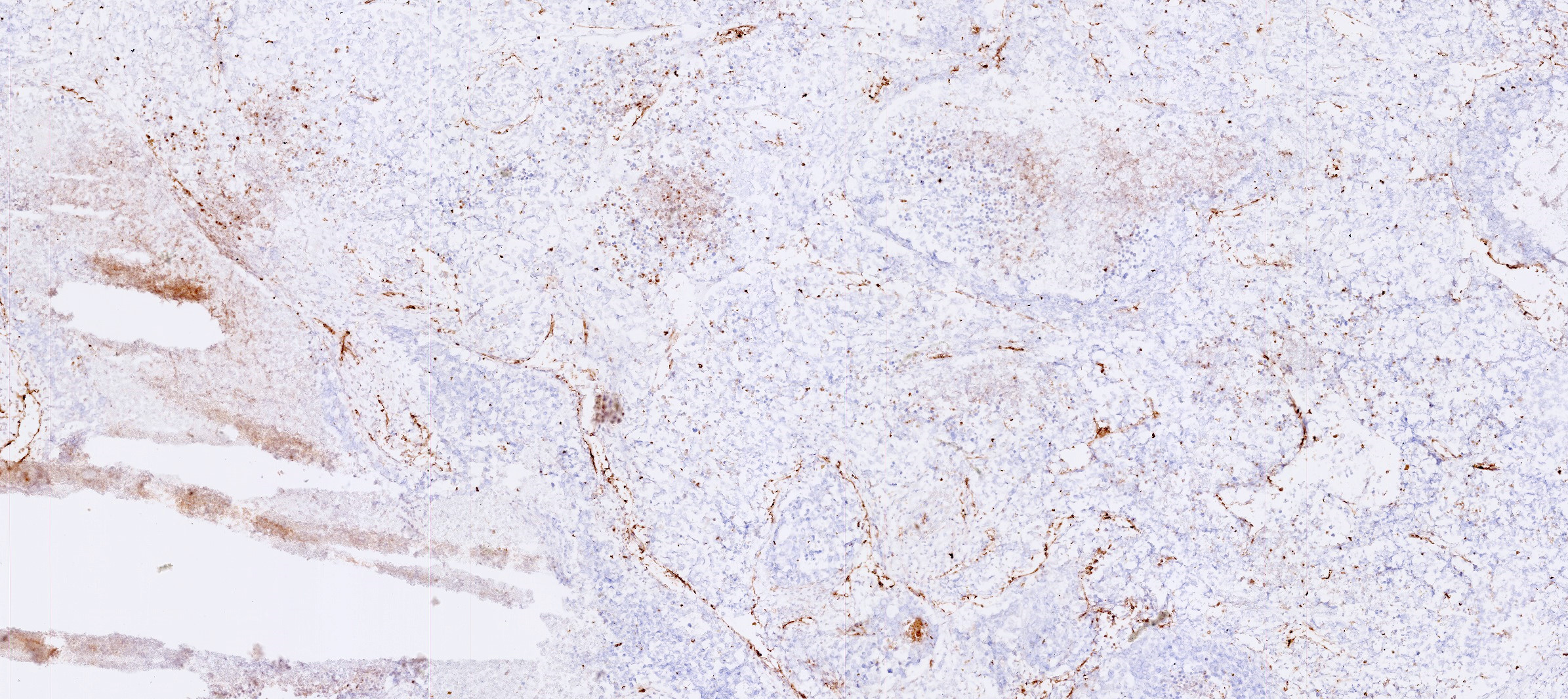

Supplement: Supplementary file 2 — Source Data for Expanded View [file EMMM-13-e11902-s002.zip › EMM-2019-11902_V3_SourceDataForEV2A-E/FigEV2C_10B3.jpeg]

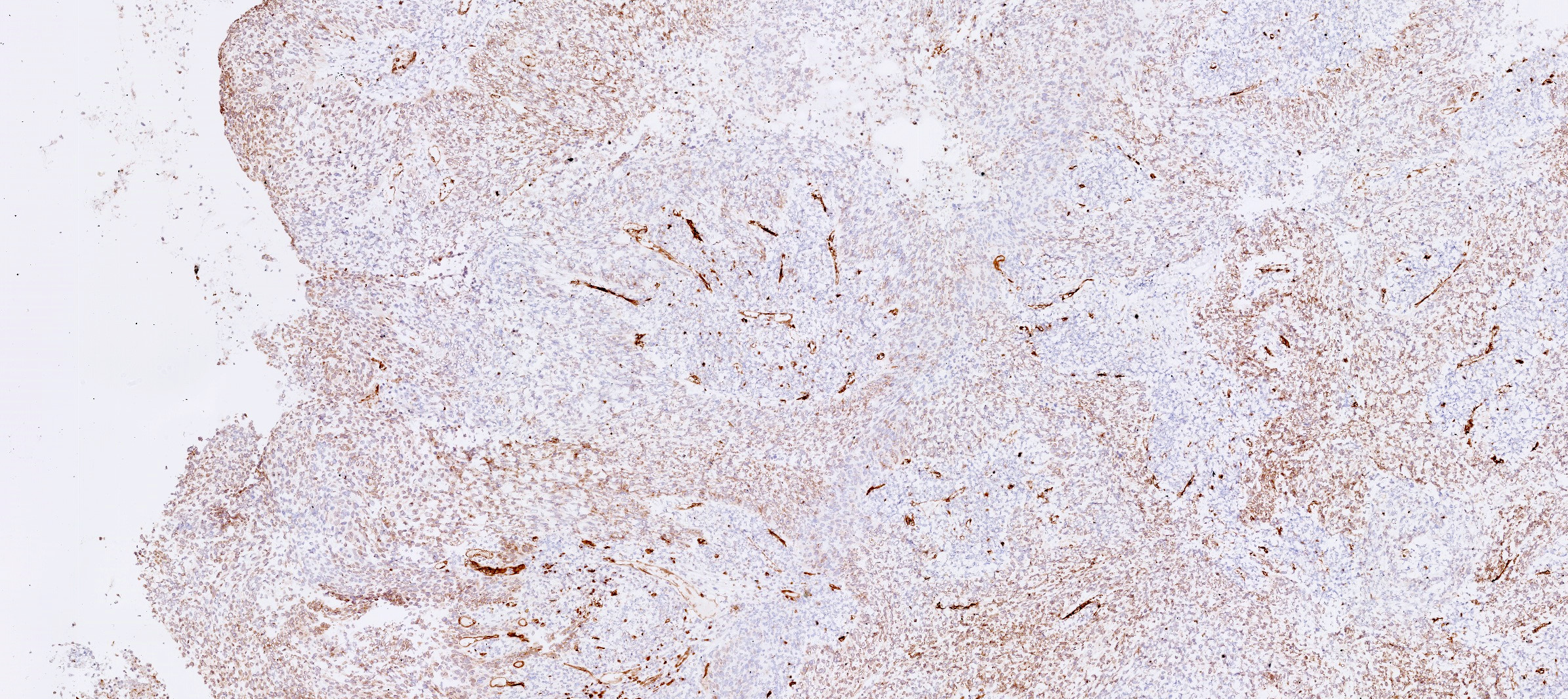

Supplement: Supplementary file 2 — Source Data for Expanded View [file EMMM-13-e11902-s002.zip › EMM-2019-11902_V3_SourceDataForEV2A-E/FigEV2B_10B3.bmp]

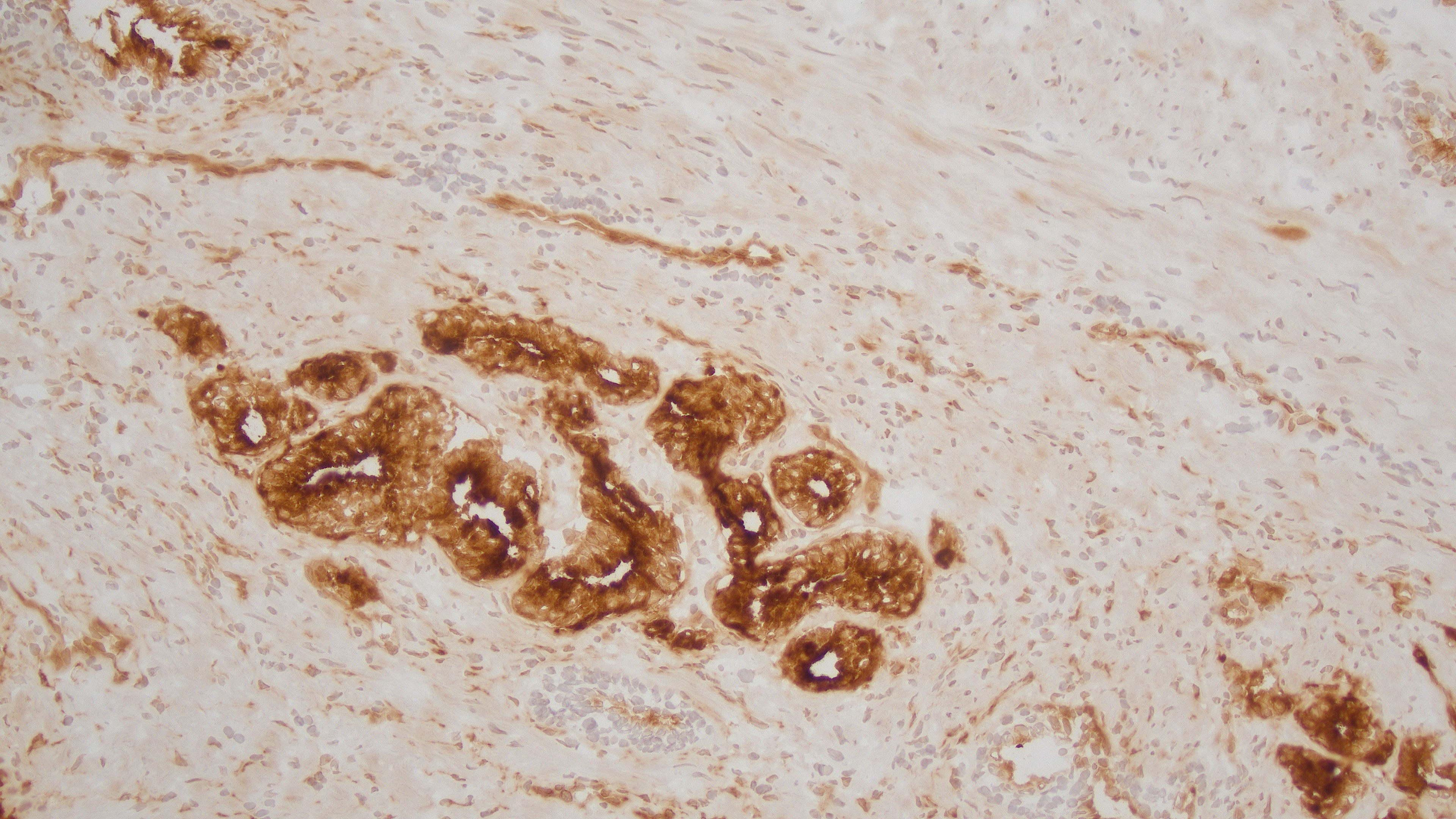

Supplement: Supplementary file 2 — Source Data for Expanded View [file EMMM-13-e11902-s002.zip › EMM-2019-11902_V3_SourceDataForEV2A-E/FigEV2A_10B3.jpg]

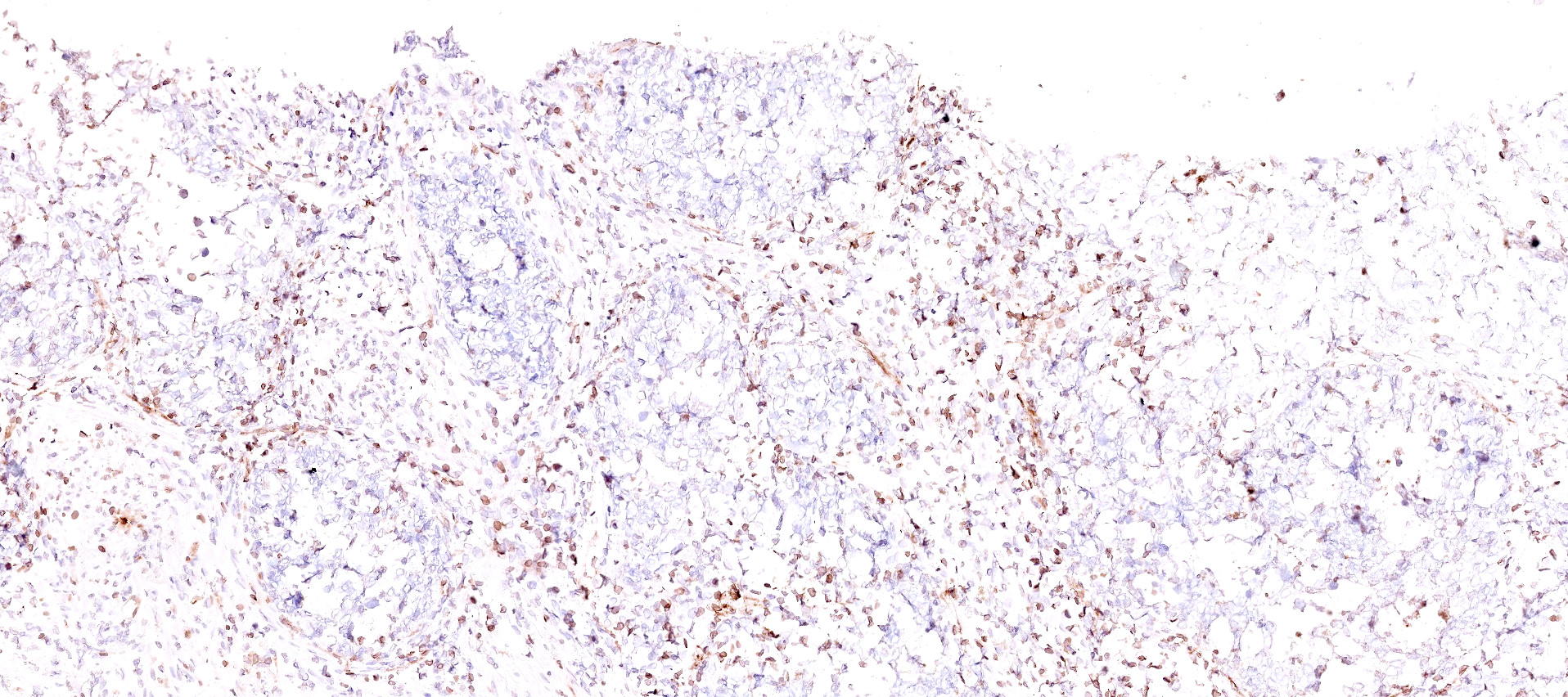

Supplement: Supplementary file 2 — Source Data for Expanded View [file EMMM-13-e11902-s002.zip › EMM-2019-11902_V3_SourceDataForEV2A-E/FigEV2D_IgGsc.bmp]

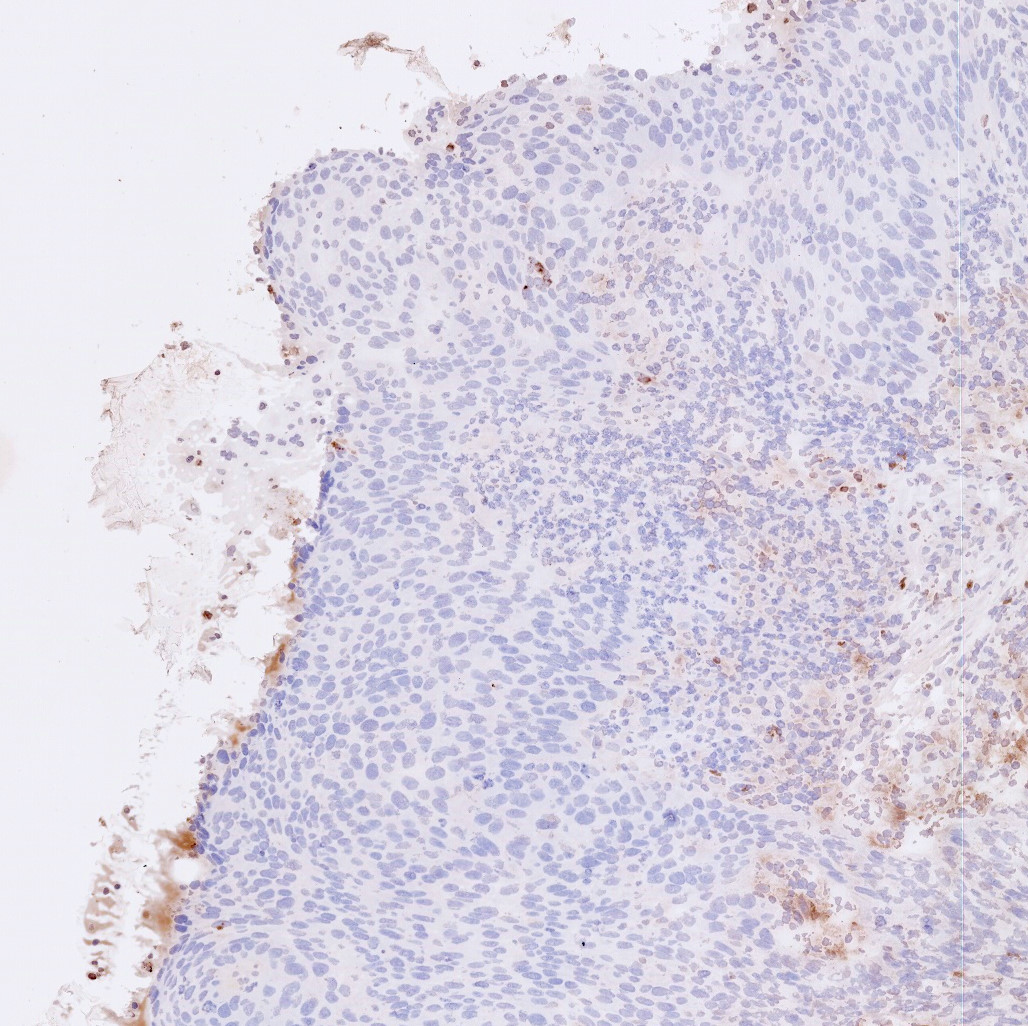

Supplement: Supplementary file 2 — Source Data for Expanded View [file EMMM-13-e11902-s002.zip › EMM-2019-11902_V3_SourceDataForEV2A-E/FigEV2E_10B3+rPSMA.bmp]

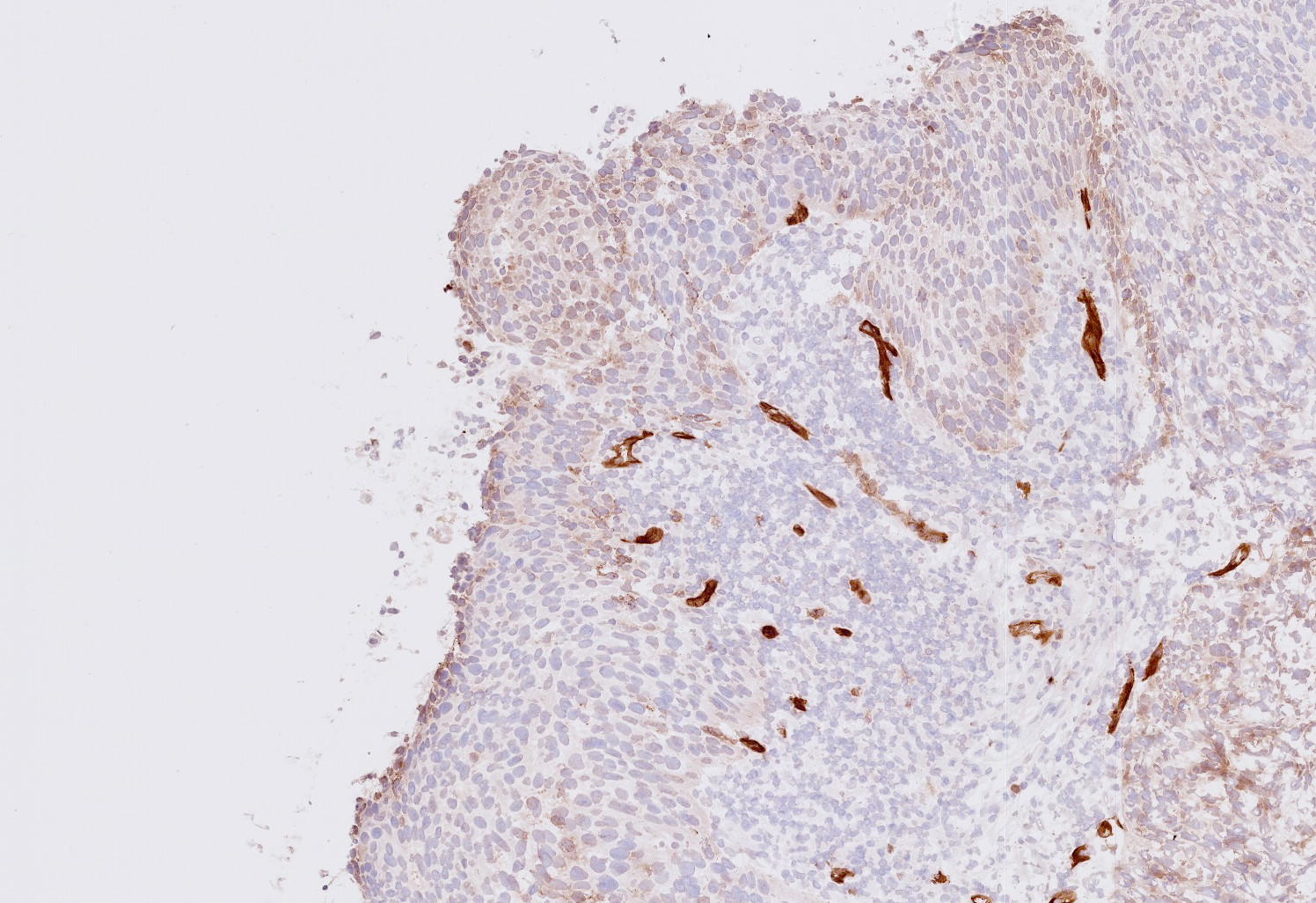

Supplement: Supplementary file 2 — Source Data for Expanded View [file EMMM-13-e11902-s002.zip › EMM-2019-11902_V3_SourceDataForEV2A-E/FigEV2E_10B3.bmp]

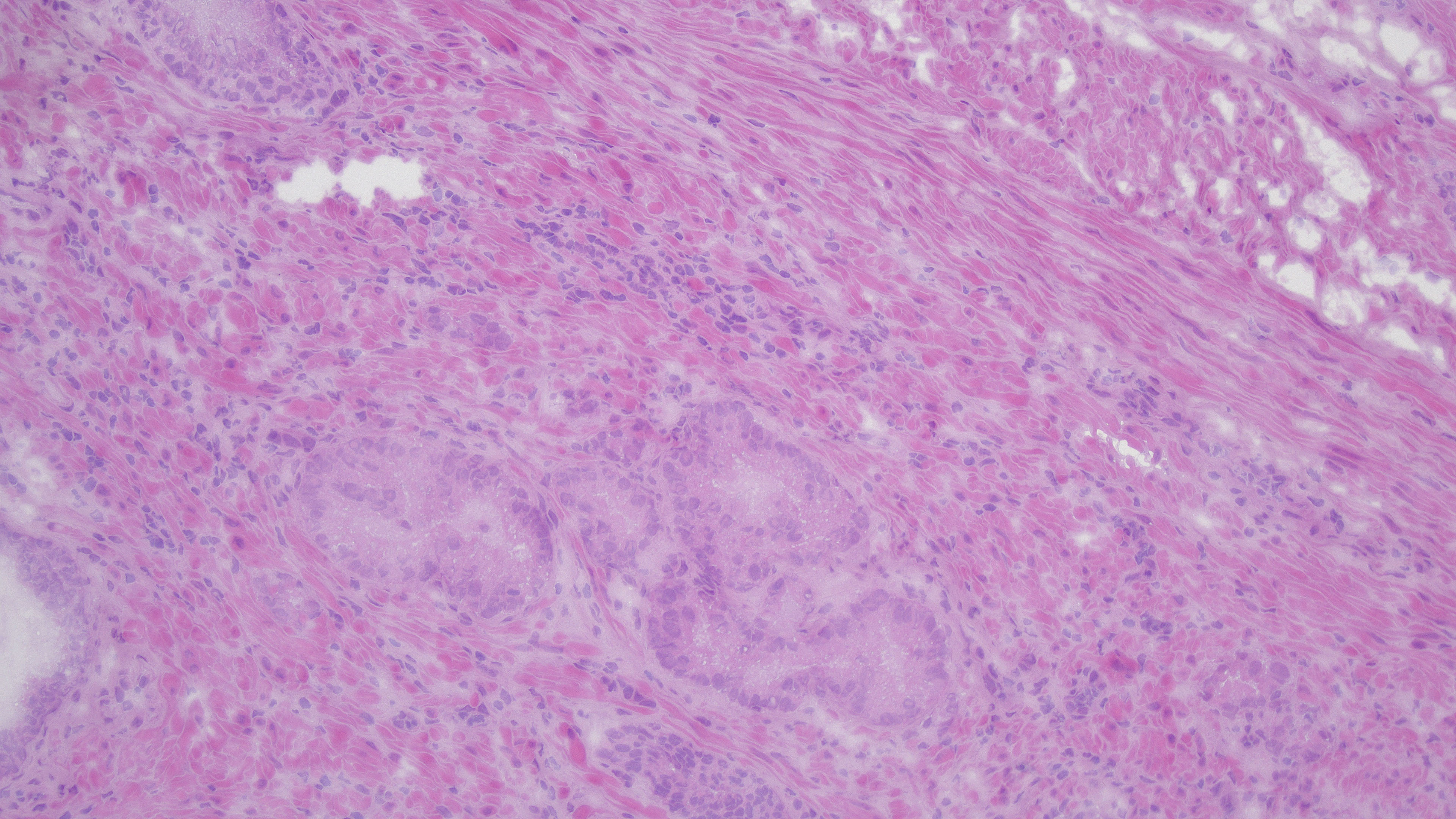

Supplement: Supplementary file 2 — Source Data for Expanded View [file EMMM-13-e11902-s002.zip › EMM-2019-11902_V3_SourceDataForEV2A-E/FigEV2A_HE.jpg]

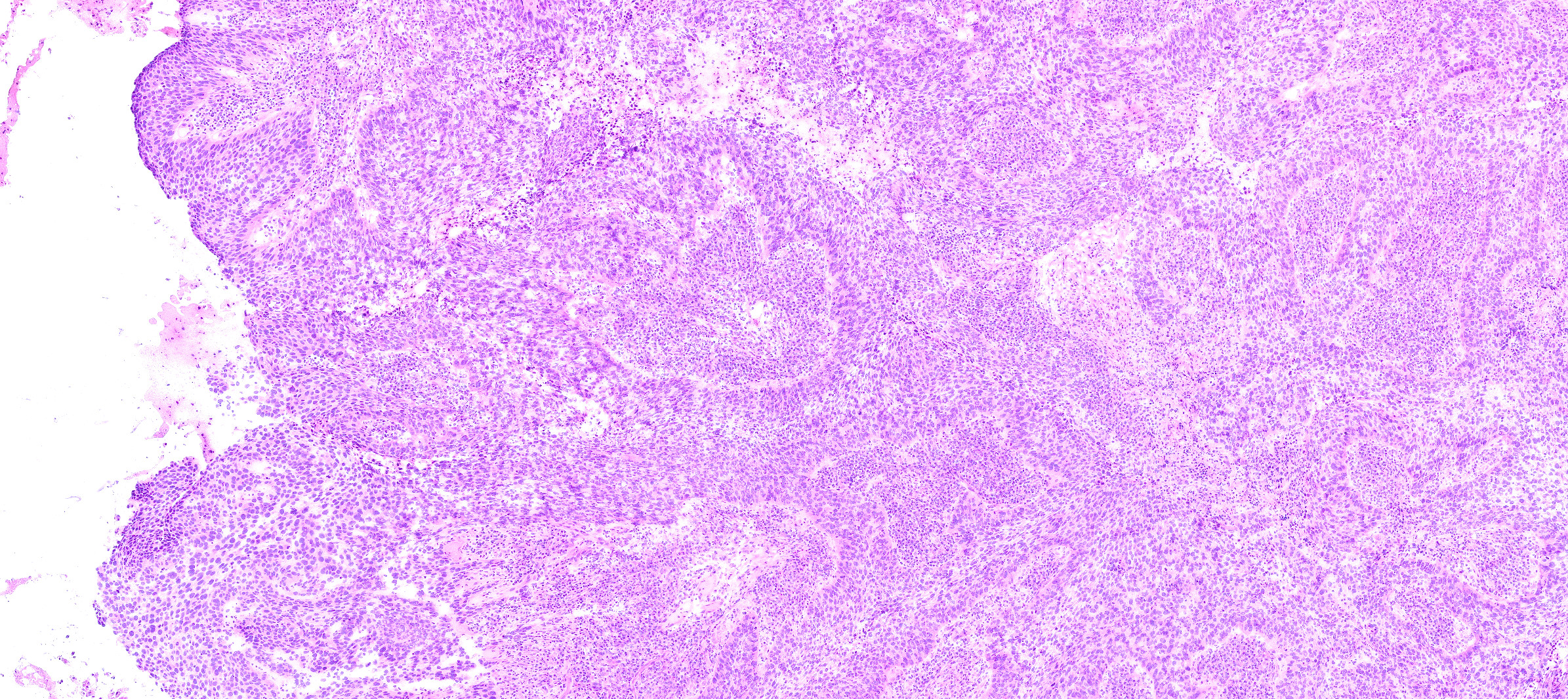

Supplement: Supplementary file 2 — Source Data for Expanded View [file EMMM-13-e11902-s002.zip › EMM-2019-11902_V3_SourceDataForEV2A-E/FigEV2B_HE.bmp]
